# Organ Donation after Open Abdominal Management with Negative-Pressure Dressing: A Case Report

**DOI:** 10.70352/scrj.cr.24-0174

**Published:** 2025-05-27

**Authors:** Hiroshi Matsumoto, Keizo Kaku, Shinsuke Kubo, Yu Hisadome, Hiroshi Noguchi, Yasuhiro Okabe, Koichi Morisaki, Keita Takahashi, Kenta Momii, Noriyuki Kaku, Tomohiko Akahoshi, Masafumi Nakamura

**Affiliations:** 1 Department of Surgery and Oncology, Graduate School of Medical Sciences, Kyushu University, Fukuoka, Fukuoka, Japan; 2 Department of Surgery and Science, Graduate School of Medical Sciences, Kyushu University, Fukuoka, Fukuoka, Japan; 3 Emergency & Critical Care Center, Kyushu University Hospital, Fukuoka, Fukuoka, Japan

**Keywords:** organ transplantation, organ donation, open abdominal management, abdominal injury, damage control surgery, ABTHERA

## Abstract

**INTRODUCTION:**

The shortage of organ donors is a major challenge in transplantation. Expanding donor eligibility criteria can help increase the donor pool, but it is crucial to carefully assess the risks related to infections in donors with expanded criteria. Organ donation from trauma patients who have undergone open abdominal management (OAM) is uncommon because of concerns regarding organ damage and infection risk. However, with appropriate OAM and stringent infection control, safe organ donation may be possible.

**CASE PRESENTATION:**

We herein present a case involving a patient who sustained abdominal organ injuries and head trauma from a fall. Emergency laparotomy was performed, including splenectomy for a splenic injury and liver laceration repair, followed by OAM using ABTHERA (3M Health Care, St. Paul, MN, USA). The patient subsequently developed irreversible brain damage and was declared brain dead. The patient’s family consented to organ donation. Following thorough evaluation, the heart, lungs, and liver were successfully recovered and transplanted into recipients at three different institutions, with no severe infections or rejection episodes reported.

**CONCLUSIONS:**

This case illustrates that with proper management using ABTHERA in OAM, organ donation can be safely achieved even in challenging cases involving trauma patients.

## Abbreviations

BVPT
: Baraker’s vacuum packing technique
CT
: computed tomography
OAM
: open abdominal management
VV ECMO
: veno-venous extracorporeal membrane oxygenation

## INTRODUCTION

The shortage of organ donors is a critical issue both in Japan and globally. Despite the increasing demand for organ transplantation, donor availability remains insufficient.^1)^ Expanding donor eligibility could help address this problem. Reports indicate that transplantation from donors with active infections, such as sepsis, can be performed safely if appropriate antibiotic treatment is administered.^2)^ However, while there are a few reports of transplantation from donors with traumatic abdominal organ injuries,^3,4)^ only one documented case of liver transplantation from a donor who underwent open abdominal management (OAM) exists.^5)^

In the context of multiple traumas involving abdominal organ injuries, damage control surgery has rapidly gained widespread adoption in the field of acute care surgery and is associated with a reduction in early mortality rates.^6,7)^ However, infections related to abdominal contamination remain a significant challenge. Reports suggest that approximately 17.6% to 50% of patients undergoing OAM develop infectious complications such as peritonitis or wound infections.^8,9)^ In organ transplantation, using organs from donors who have undergone OAM carries the risk of transmitting these infections.

ABTHERA, developed in the early 2000s by Kinetic Concepts, Inc. (now part of 3M Health Care, St. Paul, MN, USA), is a type of negative-pressure wound therapy designed to manage abdominal wounds and prevent infections. It maintains negative pressure within the abdomen, facilitates efficient drainage, and is associated with reduced abdominal pressure, decreased visceral edema, improved tissue perfusion, and prevention of fascial contraction.^10,11)^ These effects suggest that ABTHERA may reduce the risk of infection in abdominal organs. Effective control of the infection risk associated with OAM could make organ donation possible after OAM and potentially expand the donor pool.

## CASE PRESENTATION

**Fig. 1:**
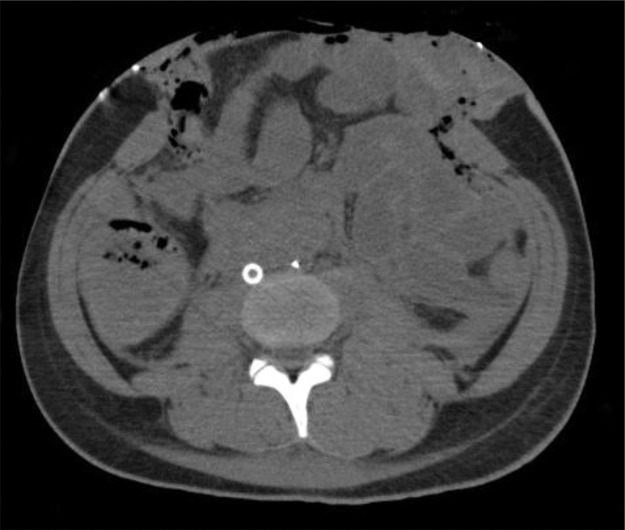
CT images after gauze packing and temporary closure using iodine drapes.

The patient was urgently transported to our hospital following a fall-related trauma. Initial evaluation with Focused Assessment with Sonography for Trauma (FAST) revealed free fluid around the spleen. Given the patient’s unstable hemodynamics, resuscitative endovascular balloon occlusion of the aorta was performed via the right femoral artery, and an emergency laparotomy was performed on-site. Following the median incision from the xiphoid process to the pubic symphysis, we identified a type IIIa+IV splenic injury, a type II liver injury, and a left retroperitoneal hematoma. Damage control surgery was initiated, including splenectomy for the splenic injury and gauze packing for hemostasis of the liver injury. Although left renal injury was suspected based on the presence of the retroperitoneal hematoma, active bleeding was ruled out as the vital signs stabilized; therefore, no intervention was performed. No bowel injury was detected, but severe bowel edema made primary abdominal closure difficult (**Fig. 1**). Thus, temporary abdominal closure was performed using iodine drapes.

Postoperatively, an initial CT scan revealed brain contusions with a midline shift, a right epidural hematoma, a left subdural hematoma, bilateral renal injuries, multiple fractures, bilateral lung contusions, and pneumothorax. Despite intubation, the patient had persistent ventilation difficulties, leading to the introduction of veno-venous extracorporeal membrane oxygenation (VV ECMO) with drainage via the right femoral vein and infusion via the right internal jugular vein, while heparin was withheld due to the risk of bleeding. Follow-up head CT performed 3 hours later showed no enlargement of the hematomas but revealed diffuse brain injury with cerebral edema, leading to the conclusion that neurosurgical intervention could potentially result in the patient becoming in an uncontrollable state. For the cerebrum, which required a conservative management approach, cerebral edema treatment was adopted.

**Fig. 2:**
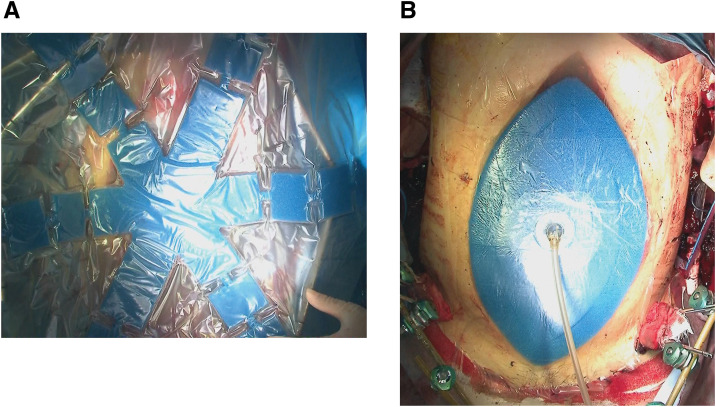
(**A**) Temporary abdominal closure using ABTHERA (3M Health Care, St. Paul, MN, USA) was performed. A protective layer was placed in the abdominal cavity to separate the abdominal wall from the organs, ensuring protection of the intra-abdominal organs and facilitating efficient drainage. (**B**) The ABTHERA blue foam was cut to the appropriate size and applied, followed by the attachment of a SENSAT.R.A.C. (3M Health Care). pad tube. This setup applied negative pressure to draw the abdominal wall inward, minimizing fascial and abdominal wall retraction. Finally, the V.A.C. drape (3M Health Care) was applied to seal the wound from the external environment.

On day 2, a second-look laparotomy was performed. Persistent bleeding from the liver injury required additional hemostasis, which was nearly complete after irrigation, followed by temporary abdominal closure with ABTHERA (**Fig. 2A**, **2B**). On day 4, the patient developed pupillary dilation, and a head CT scan confirmed brain herniation, indicating irreversible brain damage. By day 5, brainstem reflexes were lost, and clinical brain death was diagnosed. The family reported that the patient had previously expressed a desire for organ donation, and the transplant coordinator provided the necessary explanation. On day 6, ABTHERA was replaced, and intra-abdominal irrigation was performed. During this procedure, the liver, despite being repaired, appeared free of fatty liver and had a healthy color. The pancreas was salmon-pink, with no firm areas observed, although a stapler used during splenectomy was found near the pancreatic tail. Observation of the kidneys was challenging.

**Fig. 3:**
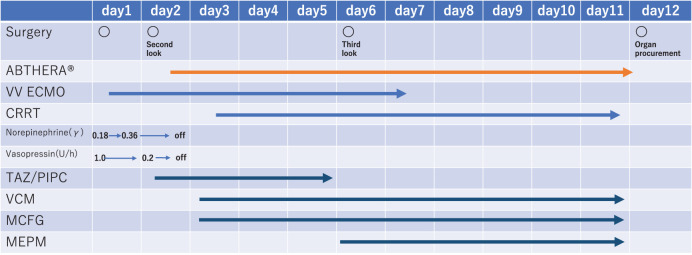
Treatment progress. CRRT, continuous renal replacement therapy; MCFG, micafungin; MEPM, meropenem; TAZ/PIPC, piperacillin/tazobactam; VCM, vancomycin; VV ECMO, veno-venous extracorporeal membrane oxygenation

Antibiotics were administered as follows: tazobactam/piperacillin from days 2 to 5, vancomycin and micafungin on day 3, and meropenem from days 6 to 12. Organ evaluation was conducted between days 6 and 10. ECMO was weaned on day 7 (**Fig. 3**).

### Overall assessment

Abdominal midline opening with ABTHERA for OAM.

### Laboratory tests (day 10)

The results are shown in **Table 1**.

**Table 1:** Laboratory tests (Day 10)

Test	Result	Units	Reference range
White blood cells	39600	/μL	4000–11000
Red blood cells	4000000	/μL	3.9–5.2 million
Hemoglobin	12.7	g/dL	12–16 (female)
Platelets	101000	/μL	150000–450000
C-reactive protein	3.28	mg/dL	<1.0
Total protein	6.5	g/dL	6.0–8.3
Albumin	3.1	g/dL	3.5–5.0
Hemoglobin A1c	5.6	%	<5.7
Creatinine	3.68	mg/dL	0.6–1.2
Blood urea nitrogen	72	mg/dL	7–20
Aspartate aminotransferase	161	U/L	10–40
Alanine aminotransferase	234	U/L	7–56
Lactate dehydrogenase	2181	U/L	125–220
Alkaline phosphatase	234	U/L	45–115
Gamma-glutamyl transferase	343	U/L	9–48
Total bilirubin	5.0	mg/dL	0.1–1.2
Direct bilirubin	3.1	mg/dL	0–0.3
Amylase	729	U/L	30–110
Lipase	467	U/L	0–160

### Cultures

Blood cultures were performed on days 3, 6, and 10, all of which were negative. Pleural fluid culture was performed on day 4, and sputum culture was performed on day 10, both of which were negative for bacteria.

### Organ evaluation

#### Kidneys

Continuous renal replacement therapy was administered from days 4 to 11. CT scan (day 1) revealed subcapsular hematomas in both kidneys with minor extravasation and poor enhancement suggested potential damage or renal infarction. Ultrasound (day 10) showed that the left kidney was 105 × 58 mm, with unclear contours and internal structure, indicating possible damage. The right kidney was 106 × 49 mm and had two subcapsular hematomas at the tail end, though it showed good blood flow and no signs of hydronephrosis.

#### Liver

**Fig. 4:**
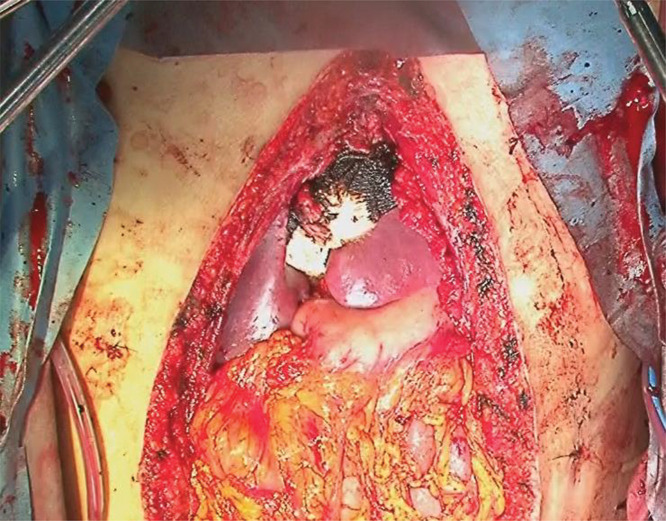
Hemostasis was achieved using sutures, TachoSil fibrin sealant patches, and SURGICEL SNoW absorbable hemostat.

CT scan (day 1) revealed damage to liver segments S3 (ventral) and S4 (portal side) with no clear extravasation and edema observed around the portal vein. Gross findings (day 6) showed type II liver damage in segments S3 and S4, and hemostasis was achieved using sutures, TachoSil fibrin sealant patch, and SURGICEL SNoW absorbable hemostat (Ethicon, Inc., Somerville, NJ, USA) (**Fig. 4**). Ultrasound (day 10) showed no clear damage to the lateral segments and right lobe; however, other areas were difficult to assess due to packing.

#### Pancreas

CT scan (day 10) indicated the presence of a stapler from the splenectomy at the pancreatic tail, which made damage assessment challenging.

#### Small intestine

CT scan (day 1) showed that intestinal blood flow was maintained and no obvious intestinal damage was observed. Gross findings (day 6) revealed no obvious intestinal damage, and no intestinal edema or dilation was observed.

#### Lungs

VV ECMO was administered from days 1 to 7. CT scan (day 1) revealed fractures in the left ribs 6, 8, 9, and sternum, hematoma extending from the neck to the anterior mediastinum, and extensive pulmonary contusions in both lungs along with left hemothorax and slight right pneumothorax. Bronchoscopy (day 10) showed brown, viscous sputum in the right upper lobe entrance, left B1–3, and B8–10, with no active bleeding. Arterial blood gas (day 11) showed pH of 7.374, partial pressure of oxygen of 147.4 mmHg, partial pressure of carbon dioxide of 39.0 mmHg, and bicarbonate of 22.6 mEq/L. The P/F ratio was 367 (fraction of inspired oxygen [FiO2]: 0.4, positive end-expiratory pressure: 10 cmH_2_O, peak inspiratory pressure: 27 cmH_2_O, respiratory rate: 16 breaths/min).

#### Heart

CT scan (day 1) revealed no calcification in the coronary arteries. Echocardiogram (day 10) showed an ejection fraction of 67.7%, with mild hypokinesis in the anterior wall and septal base. There were no global contractile issues and no valvular disease. Electrocardiogram (day 11) demonstrated normal sinus rhythm, negative T waves in V1 and V2, and no axis deviation.

#### Organ donation plan and recipient clinical course

The planned organs for donation included the heart, lungs, liver, pancreas, small intestine, and both the kidneys. Legal brain death was determined on day 10 and confirmed on day 11, finalizing the diagnosis of brain death.

Organ donation proceeded on day 12. The final organs donated were the heart, lungs, and liver. These organs were thoroughly evaluated, deemed suitable for transplantation, and subsequently shipped to their respective facilities for transplantation into recipients.

All recipients demonstrated favorable outcomes with no severe rejection reactions or serious infections, and no graft-related infections were observed in any of the transplanted organs within the first month, ensuring stable overall health.

## DISCUSSION

OAM is employed in patients who have multiple trauma with abdominal organ injuries that make abdominal closure challenging. In such cases, temporary abdominal closure methods are often used as part of damage control surgery.^6,7)^ This approach is typically used in patients with severe physiological derangements (e.g., acidosis, hypothermia, and coagulopathy), with the goal of preventing complications such as abdominal compartment syndrome while facilitating re-exploration. Abdominal closure is deferred until these physiological disturbances improve.

OAM can lead to insensible fluid loss through the abdomen and hypothermia. Such cases necessitate rigorous fluid management^12)^ and pose risks of enteric fistulas, sepsis, and intra-abdominal abscesses. Temporary abdominal closure techniques are expected to mitigate some of these complications.^6)^ ABTHERA is one such method, featuring a protective layer for efficient drainage and organ protection, a blue foam that prevents abdominal wall retraction through negative pressure, and a V.A.C. drape (3M Health Care) to cover the open wound, thus isolating, protecting, and closing the organs from the external environment. Negative pressure is applied to the wound using a SENSAT.R.A.C. (3M Health Care) pad tube, allowing for simultaneous monitoring. In vitro clinical trials have shown that the V.A.C. abdominal dressing system has significant advantages over Baraker’s vacuum packing technique (BVPT) in terms of pressure delivery, the fluid removal rate, and treatment reproducibility.^13)^ Additionally, randomized controlled trials comparing ABTHERA with BVPT have demonstrated extended survival rates.^14)^

In this case, organs (including the heart, lungs, and liver) from a patient who underwent OAM with ABTHERA were donated. It is extremely rare for organs to be donated from patients undergoing OAM because of concerns regarding infection, and reports of such cases are limited. Indeed, abdominal packing for more than 72 hours is associated with risks of sepsis and abscess formation,^15)^ and there are reported cases of infection risks in organ donation from patients who underwent OAM.^16)^ Therefore, appropriate infection control and thorough evaluation of each organ are essential when considering organ donation in these patients.

According to the World Society of Emergency Surgery guidelines, the risk factors for infection in trauma patients include the initial Sequential Organ Failure Assessment score, the Erlanghausen score, and the severity of the trauma. Regular evaluation of organ dysfunction using Sequential Organ Failure Assessment scores every 24 hours is recommended to monitor for sequential increases, which may indicate infection.^17)^ The sequential organ failure assessment (SOFA) score ranged from 11 to 14 in our case (**Table 2**). For OAM management, frequent dressing changes (washout) within 24 to 72 hours are recommended.^6)^ In this case, ABTHERA was used for OAM with dressing changes performed on day 6. Regular assessment of each organ and strict fluid management were conducted in the intensive care unit. Although peritoneal cultures were not obtained, other cultures were negative. Additionally, broad-spectrum antibiotics were used, ensuring that no fatal or severe infections compromised the transplanted organs. This allowed for successful organ donation.

**Table 2:** Progression of the sequential organ failure assessment (SOFA) score

	Day 1	Day 2	Day 3	Day 4	Day 5	Day 6	Day 7	Day 8	Day 9	Day 10
Respiration										
PaO2/FiO2 (mmHg)	204	219	194	278	368	430	266	298	337	369
Score	2	2	3	2	1	0	2	2	1	1
Coagulation										
Platelets (×10^3^/μL)	88	82	76	78	53	54	95	104	101	105
Score	2	2	2	2	2	2	2	1	1	1
Liver										
Bilirubin (mg/dL)	0.9	1.8	2.7	2.2	1.9	2.7	3.1	4.9	5.0	4.4
Score	0	1	2	2	1	2	2	2	2	2
Cardiovascular										
MAP (mmHg)	81 (NA)	88	97	99	109	81	87	99	99	102
Score	4	0	0	0	0	0	0	0	0	0
Central nervous system										
GCS	3	3	3	3	3	3	3	3	3	3
Score	4	4	4	4	4	4	4	4	4	4
Renal										
Creatinine (mg/dL)	1.25	2.84	4.11	4.06	4.65	4.83	4.39	4.00	3.68	3.60
Score	1	2	3	3	3	3	3	3	3	3
Total score	13	11	14	13	11	11	13	12	11	11

PaO2, partial pressure of oxygen; FiO2, fraction of inspired oxygen; MAP, mean arterial pressure; NA, noradrenaline; GCS, Glasgow Coma Scale.

A significant advantage in this case was the presence of liver, kidney, and pancreas transplant specialists within the hospital, enabling direct evaluation of organ conditions and the determination of organ donation feasibility. However, a limitation is that while ABTHERA may potentially reduce peritoneal and systemic inflammation by efficiently removing fluids and infectious materials from the abdomen through negative pressure and drainage,^11)^ some reports indicate that inflammatory cytokine levels in the plasma of patients treated with ABTHERA did not show a significant reduction relative to those treated with BVPT.^11)^ Additionally, in this case, peritoneal cultures were not performed, and it is unclear whether ABTHERA directly contributed to infection control. The mechanisms of infection control with ABTHERA remain unclear. Further research is needed to understand how ABTHERA reduces infection risk and whether it can safely facilitate organ donation in patients with multiple trauma and abdominal injuries. Moreover, clear criteria for assessing adequate infection control during organ donation should be established.

## CONCLUSIONS

In patients with multiple trauma and abdominal organ injuries, the use of ABTHERA for OAM may reduce the risk of complications such as infections, potentially allowing for relatively safe organ donation. This approach could contribute to the expansion of the donor pool.

## DECLARATIONS

### Funding

The authors have no funding to declare.

### Authors’ contributions

HM wrote the initial draft of the manuscript.

KK supervised the writing of the manuscript.

SK, HN, and YO contributed to the interpretation of the findings and critically reviewed the manuscript.

KK and KM performed the surgery and critically reviewed the manuscript.

KT, KM, NK, and TA performed intensive care and critically reviewed the manuscript.

MN supervised the entire process.

All authors reviewed and approved the final manuscript.

### Availability of data and materials

Not applicable to this paper as no datasets were generated during this study.

### Ethics approval and consent to participate

Not applicable.

### Consent for publication

Due to the patient’s death, it was not possible to obtain consent from the individual, and utmost consideration was given to maintaining anonymity.

### Competing interests

The authors have no conflicts of interest to disclose.
